# RRAM-based CAM combined with time-domain circuits for hyperdimensional computing

**DOI:** 10.1038/s41598-021-99000-w

**Published:** 2021-10-06

**Authors:** Yasmin Halawani, Dima Kilani, Eman Hassan, Huruy Tesfai, Hani Saleh, Baker Mohammad

**Affiliations:** System-on-Chip Center (SoCC), Department of Electrical and Computer Engineering, Khalifa University, Abu Dhabi, UAE

**Keywords:** Engineering, Electrical and electronic engineering

## Abstract

Content addressable memory (CAM) for search and match operations demands high speed and low power for near real-time decision-making across many critical domains. Resistive RAM (RRAM)-based in-memory computing has high potential in realizing an efficient static CAM for artificial intelligence tasks, especially on resource-constrained platforms. This paper presents an XNOR-based RRAM-CAM with a time-domain analog adder for efficient winning class computation. The CAM compares two operands, one voltage and the second one resistance, and outputs a voltage proportional to the similarity between the input query and the pre-stored patterns. Processing the summation of the output similarity voltages in the time-domain helps avoid voltage saturation, variation, and noise dominating the analog voltage-based computing. After that, to determine the winning class among the multiple classes, a digital realization is utilized to consider the class with the longest pulse width as the winning class. As a demonstrator, hyperdimensional computing for efficient MNIST classification is considered. The proposed design uses 65 nm CMOS foundry technology and realistic data for RRAM with total area of 0.0077 mm^2^, consumes 13.6 pJ of energy per 1 k query within 10 ns clock cycle. It shows a reduction of ~ 31 × in area and ~ 3 × in energy consumption compared to fully digital ASIC implementation using 65 nm foundry technology. The proposed design exhibits a remarkable reduction in area and energy compared to two of the state-of-the-art RRAM designs.

## Introduction

**Figure 1:**
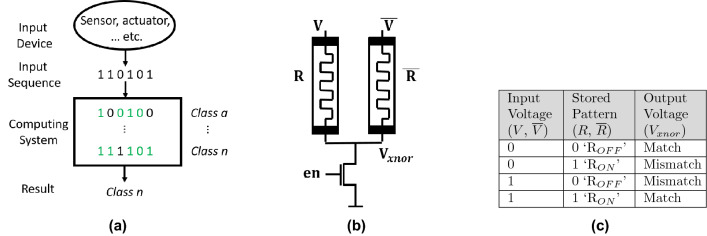
(**a**) Example of a computing system that naturally realizes in-memory search and match operations and determines the winning class. (**b**) A schematic of a two-input memristor VR-XNOR cell where one operand is voltage (*V*, $\overline{V}$), and the second is resistance (*R*, $\overline{R}$). $V_{xnor}$ is the output similarity voltage. The en configures the mode of operation of the XNOR cell. When en = 1 V, the cell is in write mode; otherwise, it will be in read mode. (**c**) Presents the associated XNOR Truth Table.

Content addressable memory (CAM) is an attractive hardware solution for applications that significantly rely on high-speed search, match, and retrieve operations^1–4^. A CAM directly performs the search within its pre-stored content in a parallel fashion with potential single cycle access, naturally realizing in-memory computing (IMC)^5,6^. As demonstrated in Fig. 1a, a CAM takes an input query and compares it against all stored patterns in a parallel manner, and returns the winning class. The traditional CAM consists of an SRAM as the memory element, which holds the pre-stored encoded data integrated with a comparator. Such design follows the pre-charge evaluate search process, which causes high power consumption and area overhead^7^. If a single mismatch occurs, the match line (ML) will discharge, and it will only stay high when all bits are matched.

As a consequence, IMC designs utilizing emerging non-volatile nano-devices for search and match operations are currently widely explored, especially for resource-constrained platforms. Resistive-CAM implementations utilize logic gates for Hamming distance computation. There are several family classifications of resistive memory-based designs depending on the input/output data representations and the underlying computational operation. When both inputs are resistance type, usually the design is used for content retrieval applications where both operands are stored in the memory^3,8,9^. While in case one operand is voltage and being compared to the second operand stored as resistance, it will benefit real-time applications for query where one vector (voltage) need to be matched with semi-static data (RRAM)^1,2,10,11^.

Other main CAM/TCAM designs reported in the literature with different structures and operational processes. For example, authors in^12^ are proposing the usage of 2T2MR-CMOL (CMOS+Molecular) architecture to increase density and reduce energy consumption along with a novel scheduling method. While in^1^, authors proposed multi-level memory cells in the design of CAM-based reconfigurable architecture. Each cell consists of a 6T2R structure to represent the three bits with two search lines (SL) and one ML. The basic cell was proposed by^11^ where both operands are analog values. The two memristor branches set the upper and lower bounds of an interval. There are two discharging paths: one to indicate a mismatch and discharges the ML to the ground, and the other path from the high SL to the low SL indicating a match since the ML stayed high. Area and energy savings were improved at the expense of increased latency by less than 20% due to digital-to-analog conversions. Another type of AM is RASSA with a 2T1R bitcells structure and depends on discharging the ML which consumes a lot of power^4^. The outputs of RASSA are locations on the reference input sequence, where alignment may result in a high score. Other non-volatile devices have been utilized, such as ferroelectric^10^, where their proposed CAM can store 3 bits in a cell using one FeFET and three FinFETs per cell. In addition, researchers in^13^ presented a PCM-based in-memory hyperdimensional computing (HDC) inference through dot-product operation. During the search operation, two crossbars are required, one to hold the data and the other to hold its complement. The part of the query hypervector is combined individually with the corresponding parts from each class by a series of AND gate arrays. Then, the resulting subvectors are fed to a series of binary adder trees, which outputs a 10-bit number representing the number of logical ‘1’ elements of the AND result per each class. These outputs are then class-wise accumulated in parallel inside the sum buffer over a period of 10 cycles. After iterating through all the partitions, a winner-take-all (WTA) circuit residing at the output of the sum buffer compares the accumulated values and outputs the index of the class with maximum accumulated value as the predicted index. Furthermore, the aforementioned prior TCAM/ACAM designs work on a two-phase-search (pre-charge) principle and incur high energy and latency overheads. Thus, CAM with computational operations based on designing static architectures for search and match are required. In^14^, the authors proposed to perform the Hamming distance calculations based on dot product operations between the input voltages and the stored conductance patterns. In such an arrangement, the only case significantly contributing to the output current is the 1 1 matching case, as demonstrated. The mismatch case 1 0 contributes slightly to the matching output current. Also, the other two cases subtract from the output since the current is flowing in the opposite direction. Hence, the logic of Hamming distance operation can be challenging with such an approach.

In this work, the focus is on the voltage-resistance input operands representation and expand on a static CAM cell design that depends on the XNOR/XOR gate that has been proposed by our group and is suitable for search index^2^. It ensures a proper computational performance of a match/mismatch operation by utilizing two memristor devices per cell as demonstrated in Fig. 1b. According to Fig. 1b, a match occurs when a low voltage, logic ‘0’, is applied to high resistance ‘R$_{OFF}$’ so the other pair will receive high voltage, logic ‘1’, on its low resistance ‘R$_{ON}$’. The produced output voltage will be high in this case. In comparison, a mismatch happens when low voltage is applied at a high conductance terminal and/or vice versa. This is based on the truth table of an XNOR logic gate as in Fig. 1c.

In this paper, a multi-bit XNOR-based RRAM-CAM is utilized for Hamming distance CAM design. It is followed by an efficient analog time-domain adder that is composed of voltage-to-time converters (VTC) and time-to-voltage converters (TVC). The design uses brain inspired HDC computing as a demonstrator. In such classification application, input data is large and is compared with a large amount of stored data in the associative memory simultaneously, where the inputs are in thousands of bits length. This raises the demand for a high-density, low-power solution.

**Figure 2:**
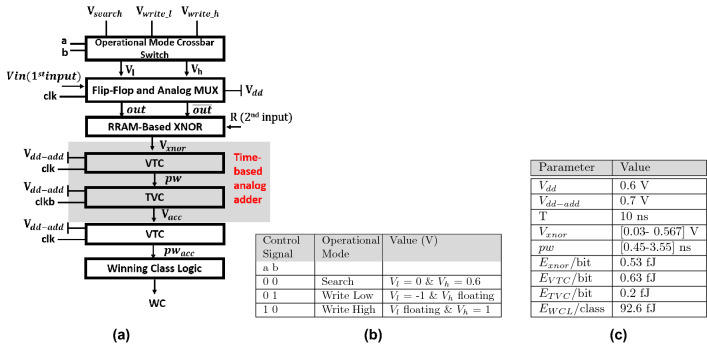
(**a**) Proposed time-domain RRAM-CAM Hamming distance and winning class data-flow block. (**b**) Proposed Design Operational Modes and Control Signals. (**c**) Proposed Architecture Design Parameters and Energy Consumption.

The proposed Hamming distance AM data-flow is shown in Fig. 2a. The flow starts by choosing the RRAM-CAM operational mode by using a 2$\times$2 crossbar switch to determine the voltages associated with each mode^15^. The XNOR-based RRAM-CAM cell has two operational modes: write and search with the control signals and corresponding values of $V_{l}$, and $V_{h}$ presented in Fig. 2b. During the writing step, memristor devices storing logic ‘0’ (R$_{OFF}$) are programmed by applying a negative voltage at its terminal while keeping it’s other pair floating. Then, a high programming voltage is applied to the devices that shall store logic ‘1’ (R$_{ON}$) while the other devices are grounded. Hence, the writing mode takes two clock cycles. It is worth mentioning that writing to the memristor devices occurs only once and stay constant throughout the lifetime of the system. This is critical as RRAM has limited endurance and for IMC-CAM application there is no need to do many writes.

When search mode is activated, the received input passes through the flip-flops to the analog MUX at each clock cycle. The analog MUX then passes a pair of high ($V_{h}$) and low ($V_{l}$) output voltages for each input bit from the sequence based on the input signal logic. The ($V_{h}$) and ($V_{l}$) are selected to be less than the write voltage of the RRAM cell to ensure minimum state disturbance to the stored value. Moreover, the direction of the current through these RRAM devices changes depending on the input sequence and hence, can adjust any small shift in the programmed value which might be caused by the search operation. These pairs of voltages now serve as the first input operand to the XNOR-based RRAM-CAM that stores the second input operand as pairs of resistance values. The output voltage from each column reflects the similarity between the input query and stored data. Each column output voltage in the crossbar is converted to a time-domain pulse using VTC to be added with other output voltages from other arrays. The produced pulse widths will be combined in time-domain and then passed to a digital logic circuit to determine the winning class with the longest pulse width. In the following subsections, a detailed discussion of the proposed circuits is presented.

## Results

### XNOR-based RRAM

**Figure 3:**
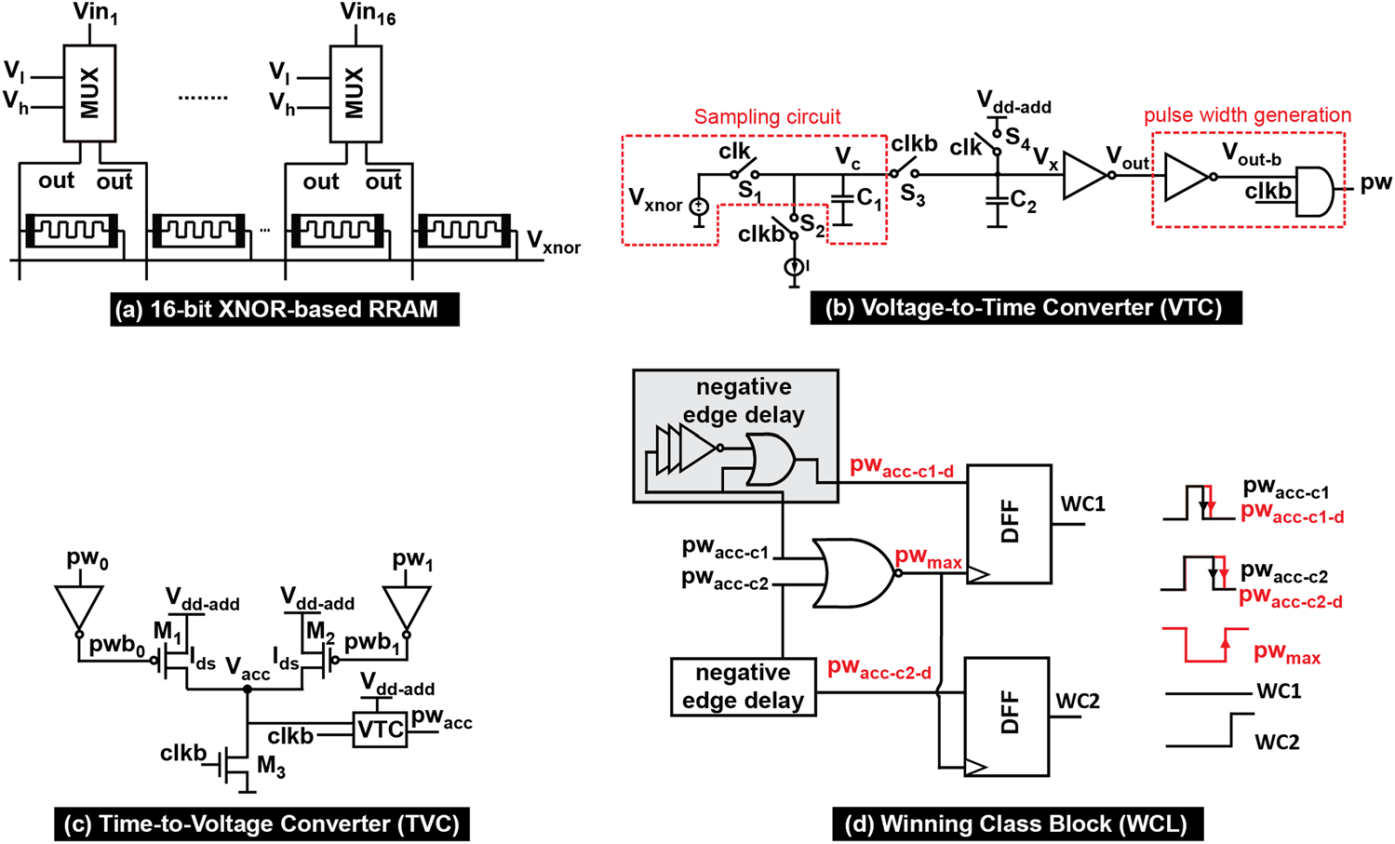
Circuit designs of the proposed RRAM-based CAM, analog time-domain adder and winning class logic. (**a**) 16-bit XNOR-based RRAM, (**b**) proposed VTC circuit, (**c**) TVC circuit and (**d**) digital winning class logic.

Figure 3a shows the 16-bit XNOR-based RRAM cell. Programming the RRAM devices to ‘R$_{OFF}$’, and ‘R$_{ON}$’ occurs only once through writing mode. The NMOS transistor acts as a switch that is ON during programming phase to ensure a path to ground, and OFF during search phase. During search mode, the value of *Vin* enables either an output of high voltage *out* or low voltage $\overline{out}$ using analog MUX. When *Vin*=0, *out*=$V_{l}$ and $\overline{out}$=$V_{h}$. On the other hand, when *Vin*=1, *out*=$V_{h}$ and $\overline{out}$=$V_{{l}}$. Assume that *out* is connected to ‘R$_{ON}$’ whereas $\overline{out}$ is connected to ‘R$_{OFF}$’. This means that when *Vin*=1, both inputs of voltage and resistance are matched resulting in an output voltage $V_{xnor}$=1 to realize an XNOR operation.

**Figure 4:**
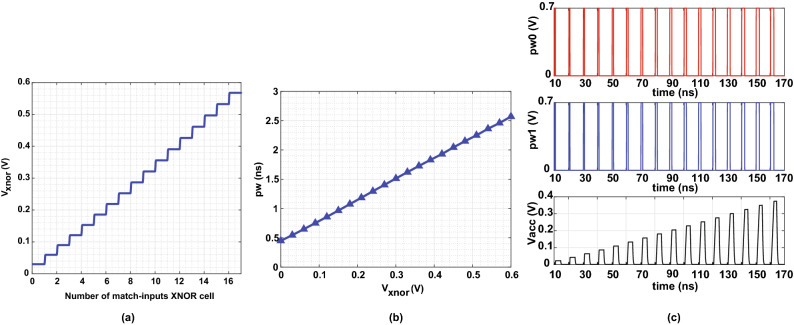
(**a**) Similarity output voltage $V_{xnor}$ versus the number of matching inputs for 16-bit XNOR-based RRAM at $V_{dd}$=0.6 V. (**b**) Modulated pulse width signal *pw* scales linearly with the similarity output voltage $V_{xnor}$ at $V_{dd-add}$=0.7 V. (**c**) Modulated pulse width signals $pw_{0}$ nd $pw_{1}$ are converted to $V_{acc}$ via TVC for a 32-bit XNOR-based RRAM divided into two 16-bit XNOR blocks. Each pulse width corresponds to number of matching-inputs XNOR cells. The minimum pulse width corresponds to all mismatching inputs and the maximum pulse width means 16 matching inputs XNOR cells.

The 16-bit XNOR-based RRAM is designed and implemented in 65 nm CMOS technology with supply voltage $V_{dd}$=0.6 V, $V_{l}$=0 V and $V_{h}$=0.6 V, and resistance values ‘R$_{ON}$’=50 k${\Omega }$ and ‘R$_{OFF}$’=1 M${\Omega }$. The XNOR-based RRAM cell is deployed using a voltage threshold adaptive memristor (VTEAM) model which is widely utilized in the literature^16^. The fitting parameters of the memristor cell using VTEAM model are selected from our previous work in^17^ and are presented in supplementary Table S1. It is worth noting that the simulations’ values were chosen based on reported real devices that can achieve acceptable noise margin and distinction between matching and mismatching cases^18^. Figure 4a shows the output voltage level of 16-bit XNOR-based RRAM versus the number of matching-inputs XNOR cells. As the number of matching-input cells increases, $V_{xnor}$ increases linearly. The value of $V_{xnor}$ increases by ~ 30 mV per one matching cell. Note that when all inputs mismatch, $V_{xnor}$ = 30 mV, whereas it reaches the maximum voltage of 0.567 V when all 16 XNOR cells are input-matched. Table S2 presents trade-offs between the number of XNOR cells per row, noise margin, and current consumption. 16-bits were chosen as they provide a good balance between noise margin and power. Moreover, Fig. S1 shows the XNOR-based RRAM histogram mismatch variations when all 16 XNOR cells are input-matched and RON and ROFF values are varied by +10%. Adding more XNOR cells saturates the output $V_{xnor}$ and will not reflect the matching inputs’ correct similarity. One possible way to address the voltage saturation issue is to operate the XNOR-based RRAM cells at a higher supply voltage that grants a larger number of bits. For example, if $V_{h}$ and $V_{dd}$ are increased to 1.2 V, the number of XNOR-based RRAM cells can be expanded to up to 32. Nonetheless, such a method adds significant power overhead to the design. Hence, a more efficient approach is to split the large XNOR-based RRAM array into *K* smaller blocks^19^. For instance, the 32-bit RRAM cells are divided into two 16-bit cells while operating at lower $V_{dd}$ that guarantees power saving. The drawback comes again when the output voltages of the *K* XNOR-based RRAM blocks saturates. In this paper, we propose a time-domain adder with analog inputs using a novel VTC discussed in the following section. Processing in the time-domain has several advantages over the voltage-domain. Both time and capacitance scale better with technology than voltage. Besides, processing in the time-domain will have less variations and high noise immunity, unlike in the analog-domain where the signal-to-noise ratio is degraded due to voltage scaling^20^.

### Time-based analog adder

The proposed time-domain adder consists of two blocks: VTC and TVC. The VTC circuit will convert $V_{xnor}$ to a modulated pulse width signal *pw*. Then, the TVC adds up all the modulated pulse width signals from the *K* 16-bit XNOR-based RRAM and converts them into a single accumulated voltage $V_{acc}$ which can then be transformed again to time via VTC and the final stage will use time-to-voltage converter to get the corresponding final pulse width.

The proposed VTC circuit, shown in Fig. 3b, is implemented and simulated in 65 nm CMOS technology at a supply voltage level $$V_{dd-add}$$=0.7 V and a frequency of 100 MHz. Pass gates replace both $$S_{1}$$ and $$S_{3}$$, whereas NMOS and PMOS transistors, respectively, replace $$ S_{2}$$ and $$S_{4}$$. The current source is implemented using an NMOS transistor that operates in the saturation region.

Figure 3b shows the block diagram of the proposed VTC circuit design. It consists of a sampling circuit, an inverter, and a current source. The $V_{xnor}$ voltage is the VTC’s input converted to a *pw* signal. In order to achieve voltage-to-time conversion, the VTC has two operating phases: sample and evaluate. During the sampling phase: $S_{1}$ and $S_{4}$ turn on when the clock *clk* is logic high and $S_{2}$ and $S_{3}$ are off when the inverted clock *clkb* is logic low. The capacitor $C_{1}$ is precharged with a voltage $V_{c}$ equals to the input voltage value $V_{xnor}$. The capacitor $C_{2}$ is charged with a voltage $V_{x}$ equals to the supply voltage $V_{dd-add}$. During the evaluation phase: $S_{1}$ and $S_{4}$ turn off when the clock *clk*=0 and $S_{2}$ and $S_{3}$ turn on when *clkb*=1. The node $V_{c}$ is coupled to $V_{x}$.The initial charge across the capacitors is $Q_{i}$=$V_{xnor}C_{1}$+$V_{dd-add}C_{2}$. Due to the potential difference between $C_{1}$ and $C_{2}$, the charges are shared among them. Consequently, the current flows from $C_{2}$ to $C_{1}$ causing a voltage pump on $V_{c}$. Then, it starts discharging through the current source *I* till it reaches the switching point of the inverter $V_{sp}$ resulting in a final charge $Q_{f}$=$V_{sp}(C_{1}+C_{2})$. After that, the inverter pulls up the delayed output voltage $V_{out}$. The time it takes to discharge $V_{x}$ to the inverter’s switching point voltage to switch from low to high is referred to as time delay $t_{d}$. This time delay, given in Eq. (1), depends on four main parameters: voltage values of $V_{dd-add}$ and $V_{xnor}$, voltage value of $V_{sp}$, capacitors’ size of $C_{1}$ and $C_{2}$ and the average current $I_{avg}$ until it is discharged.

1 $$\begin{aligned} t_{d}=\frac{Q_{i}-Q_{f}}{I_{avg}}= \frac{C_{1}V_{xnor}+C_{2}V_{dd-add}-V_{sp}(C_{1}+C_{2})}{I_{avg}} \end{aligned}$$

The inverter chain whose output $V_{out-b}$ is ANDED with *clk* to generate *pw*. The $V_{sp}$ value is set by the aspect ratio of PMOS and NMOS transistors of the inverter. The $I_{avg}$ value depends on the amount of charges stored in the capacitors which varies linearly with $V_{xnor}$ given that $V_{dd-add}$ is fixed. Thus, $t_{d}$ has a linear relationship with $V_{xnor}$. Figure 4b shows *pw* versus $V_{xnor}$. Note that *pw* scales linearly with $V_{xnor}$, and it has a gain of 3.55 ns/V and power consumption of 1.1$\mu$W.

After that, the output from the *K* VTC blocks, $pw_{k}$, is sent to the TVC circuit to generate the accumulated voltage level $V_{acc}$ that corresponds to a single class set. Figure 3c shows the TVC circuit diagram with two inputs $pw_{0}$ and $pw_{1}$ as a simple example. The $pw_{0}$ and $pw_{1}$ represent the modulated pulse width signals from the 1st and 2nd 16-bit XNOR-based RRAM cells, respectively. The inverted modulated signals $pwb_{0}$ and $pwb_{1}$ are connected to the gate of the PMOS transistors $M_{1}$ and $M_{2}$ whose sources are $V_{dd-add}$ and sizes are same. When $M_{1}$ and/or $M_{2}$ are on whereas $M_{3}$ is off, $M_{1}$ and $M_{2}$ conducts an electrical current $I_{ds}$ rising the accumulated voltage across the capacitor *C* (*C* represented by the capacitor in the VTC circuit that is needed for the WCL as shown in Fig. 3c). This voltage is linearly proportional to $pw_{0}$ and $pw_{1}$ as given in Eq. (2). As long as $M_{3}$ is off, *C* keeps holding $V_{acc}$ even when $M_{1}$ and/or $M_{2}$ are off. Once $M_{3}$ turns on when $clkb=1$, the capacitor discharges its voltage to 0 V.

2 $$\begin{aligned} V_{acc}=\frac{I_{ds}}{C}(pw_{0}+pw_{1}) \end{aligned}$$

**Figure 5:**
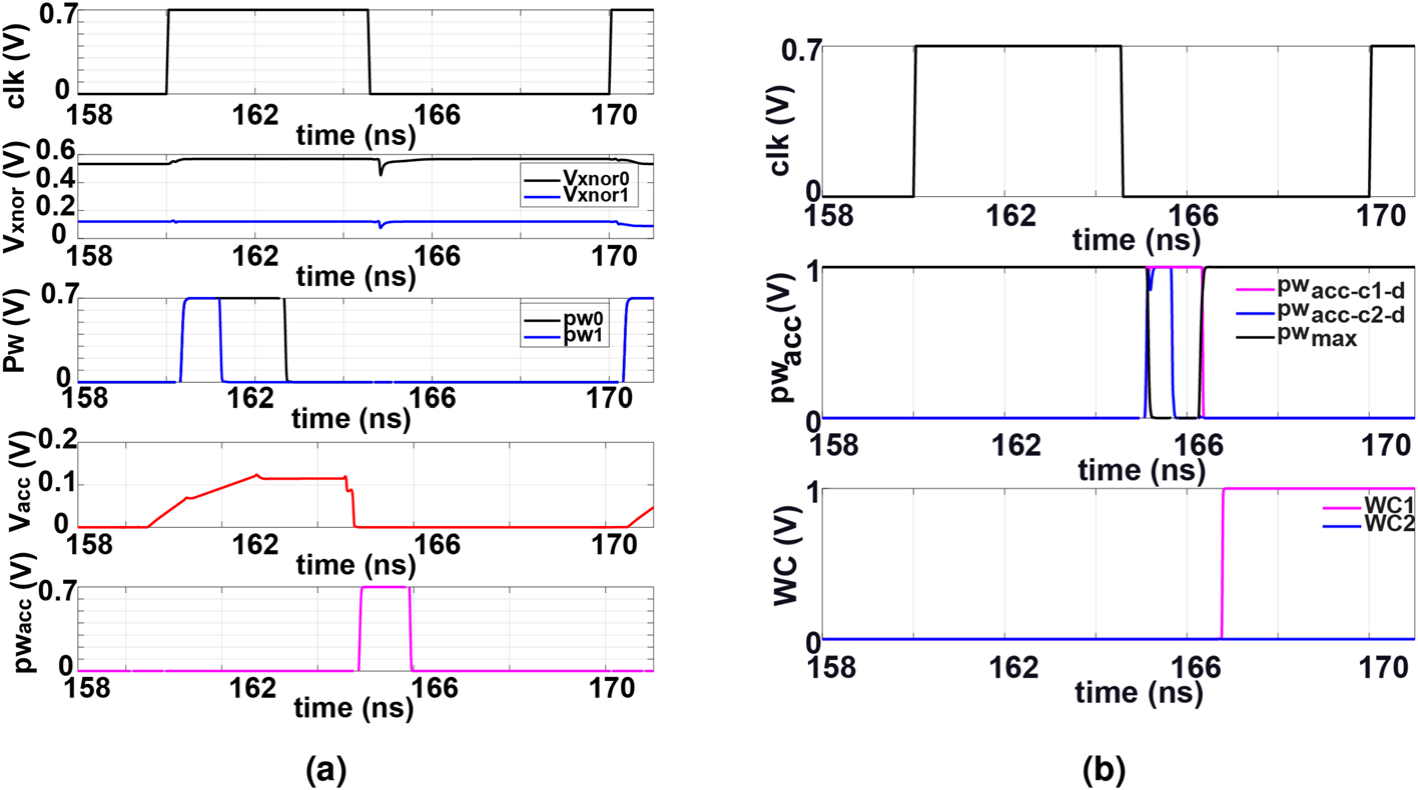
(**a**) Output waveform of the proposed block using 32-bit XNOR-based RRAM divided into two 16-bit XNOR RRAM. Each 16-bit XNOR RRAM is followed by VTC, TVC and TVC. One 16-bit XNOR RRAM with 3 matching-inputs cells and the second is with 16 matching-inputs cells. (**b**) Output waveforms of the proposed WCL block. It shows that $pw_{acc-c1-d}$ has a wider pulse width compared to $pw_{acc-c2-d}$. As a consequence, it is determined as the winning class with WC1 = 1 V and WC2 = 0 V.

The circuit can be designed to support the *K* number of *pw* as long as $V_{acc}$ does not saturate. Figure 4c depicts the output waveform of the time-domain analog adder for 32-bit XNOR-based RRAM divided into two 16-bit XNOR blocks. Figure 5a depicts the simulation result of the proposed architecture using 32-bit XNOR-based RRAM except for the WCL. As shown in the figure, the time-domain analog adder operates at the positive edge clock cycle where the VTC generates *pw* and then the TVC adds the voltage $V_{acc}$. At the negative-edge clock cycle, $pw_{acc}$ is generated using a VTC to provide the WCL. The effect of mismatch variations on the *pw* of VTC obtained from Monte Carlo simulation are presented in Figs. S2 and S3. And Table S3 shows how the variation can be reduced by cascading multiple stages of the VTC circuit.

It should be noted that although the main characteristic of HDC is its robustness to the faults associated with the computational substrates on which it is executed^21^, variations in the TVC values could pose a challenge due to the small noise margin between successively combined pulse widths. However, this can be easily addressed by reducing the number of combined pulse widths and/or increasing the voltage supply to increase the margin. Noise simulation has been carried out to analyze the input-referred noise and the SNR of the time-based analog adder whose input is $V_{xnor}$ and output is $pw_{acc}$ and results can be found in the Fig. S4.

### Winning class logic (WCL)

In order to determine the winning class, which is reflected by the maximum accumulated voltage among the multiple voltages of the different classes, a digital implementation of WCL is utilized. All the accumulated voltages from the different classes are converted to the modulated pulse width signals $pw_{acc-cn}$ (c is the class set and n corresponds to its number) using the VTC circuit and then fed to the WCL. Figure 3d depicted the circuit diagram of the WCL for two classes as a simple example. The circuit can be expanded according to the number of classes. As shown in Fig. 3d, to determine the maximum pulse width $pw_{max}$ among the two different pulse widths from two different classes $pw_{acc-c1}$ and $pw_{acc-c2}$, NOR gate is utilized. Then, $pw_{max}$ is connected to the D flip-flop (DFF) clock. Both $pw_{acc-c1}$ and $pw_{acc-c2}$ are connected to a negative-edge delay circuit whose delay is greater than the NOR gate delay to ensure setup time for the DFF. The delayed class signals $pw_{acc-c1-d}$ and $pw_{acc-c2-d}$ are connected to the D terminal of the DFF. At the positive edge of $pw_{max}$, DFF will compare between $pw_{max}$ and $pw_{acc-c1-d}$ and $pw_{acc-c2-d}$ to generate the winning class logic high while the other one remains logic low. Figure 5b shows the simulation results of the WCL block for two different classes. The signal $pw_{acc-c1}$ for class 1 has a wider pulse width compared to the signal $pw_{acc-c2}$ for class 2. This means that class 1 has a higher input similarity and hence is the winning class. Figure 2c summarizes design parameters and the energy consumption of the proposed RRAM-CAM Hamming distance architecture.

## Hyperdimensional computing architecture demonstrator

**Figure 6:**
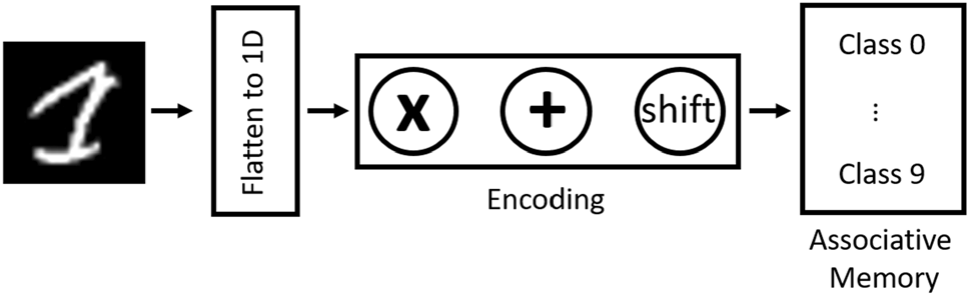
Typical HDC modules consisting of encoding and comparison. In the encoding stage, addition, multiplication, and cyclic-shifting are used to generate a single representation of hypervectors from the same class. Then encoded data is stored in the AM. During the inference phase, encoded input is applied to the AM to evaluate the closest HD class vector using the appropriate similarity metrics.

Hyperdimensional Computing (HDC) is a brain-inspired architecture by the dimensionality expansion of information processing happening in the human nervous system^19,22^. Due to the brain’s large size, the neural activity is represented in an abstract form in thousands of dimension, hyperdimensional (HD) vectors such as *d* = 1k, where *d* is the dimensionality assumed for the HD vectors. Such paradigm differs from neural network and the need to train the model for hundreds or thousands of iteration until the output converges. The HDC paradigm consists of two main modules as shown in Fig. 6: encoding and comparison for MNIST data-set classification. During the encoding phase, the following two things are created and are fixed throughout the lifetime of the system:

- Create an independent, identical distribution (i.i.d) random HD vectors for image pixel sequence representation and store them in a memory called item memory (IM). The IM size depends on the size of the image and the chosen dimension (*d*) of the HD vector. So in our case the IM will have a 784$\times$1k dimension.
- Store all encoded patterns in the associative memory (AM). For the MNIST example there are 10 classes. The AM part is used to compare the encoded query to all encoded patterns stored inside the AM, it has a dimension of 10$\times$ $d$. To be able to accommodate all the bits, we divide the matrix into 64 blocks each containing 10x16 arrays. In this paper, 1k = 1024. The input image is encoded through three operations: multiplication (binding), addition (bundling), and shifting (permutation) to transform the input to an HD vector. Also, all vectors of the same class from the training set are summed up together to generate a single representation. After that, each class’s single encoded patterns are stored in the AM for comparison during the inference phase. In our design, the encoded HD vectors are mapped into memristor conductance where ‘1’ is mapped to $G_{ON}$ and ‘0’ to $G_{OFF}$. Transfer the values into the XNOR-based RRAM-CAM array by applying specific voltage pulse to tune the conductance values.

**Figure 7:**
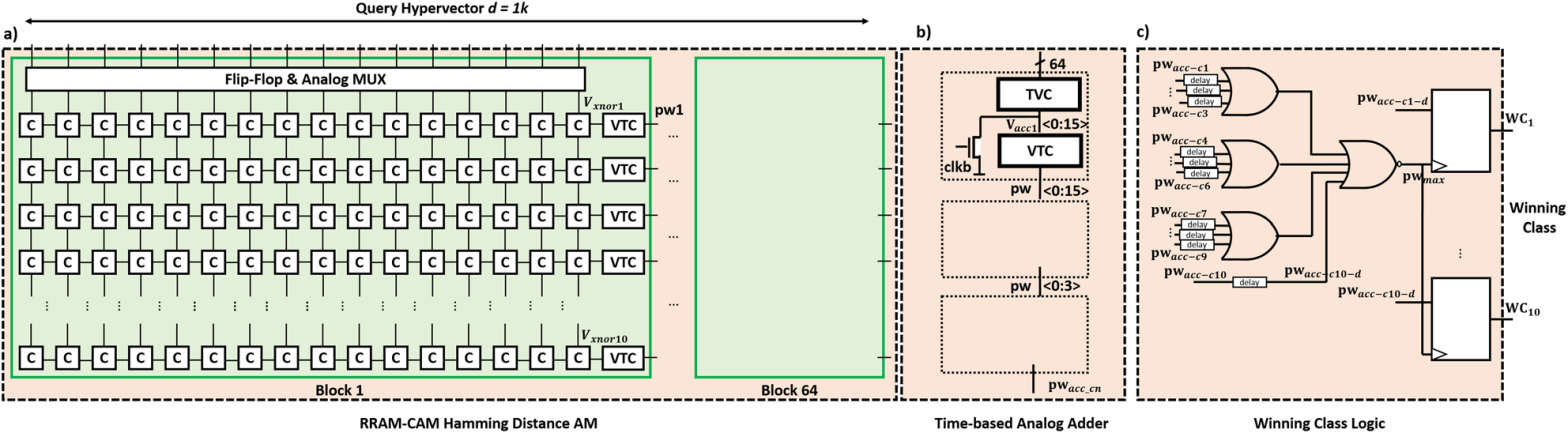
Detailed full hierarchy of the mixed-signal XNOR-based RRAM-CAM for HDC classification showing in (**a**) the 64 blocks of the divided query hypervector $10\times 16$ arrays having VTC at the end of each row, then followed by(**b**) time-based analog adder to combine entries class-wise from corresponding arrays, and eventually in (**c**) the winning class logic to decide upon the winner.

MNIST for supervised classification using orthogonal encoding using HDC paradigm has been carried out in^23^ using MATLAB. And in this work, the inference phase is considered only so the IM and the AM modules are established. The following steps are carried through the inference/testing stage:

- The first step is to flatten the $28\times 28$ to get a vector of $784\times 1$. Then, each pixel in this vector will be binarized to 0, 1 depending on its intensity and then encoded to a $1\times 1$ k HD vector. So the encoded image matrix now will have a $784\times 1$ k dimension.
- Each row in the IM will be shifted depending on the value of the $1\times 1$ k row in the encoded image matrix. If the value is 1, the IM will be shifted and stay as it is otherwise.
- Aggregate all shifted and not shifted HD vectors of the IM to generate a single HD vector representation for the image.
- Perform majority sum of the representation by adding the shifted array values column-wise and thresholding the output to binarize the HD vector. So now the query vector will have a dimension of $1\times 1$ k. This is the matrix that is used as an input in to the full system block shown in Fig. 7.
- Compare the 1D encoded binary vector to the stored representations (classes) in the AM through the Hamming distance computations.

**Figure 8:**
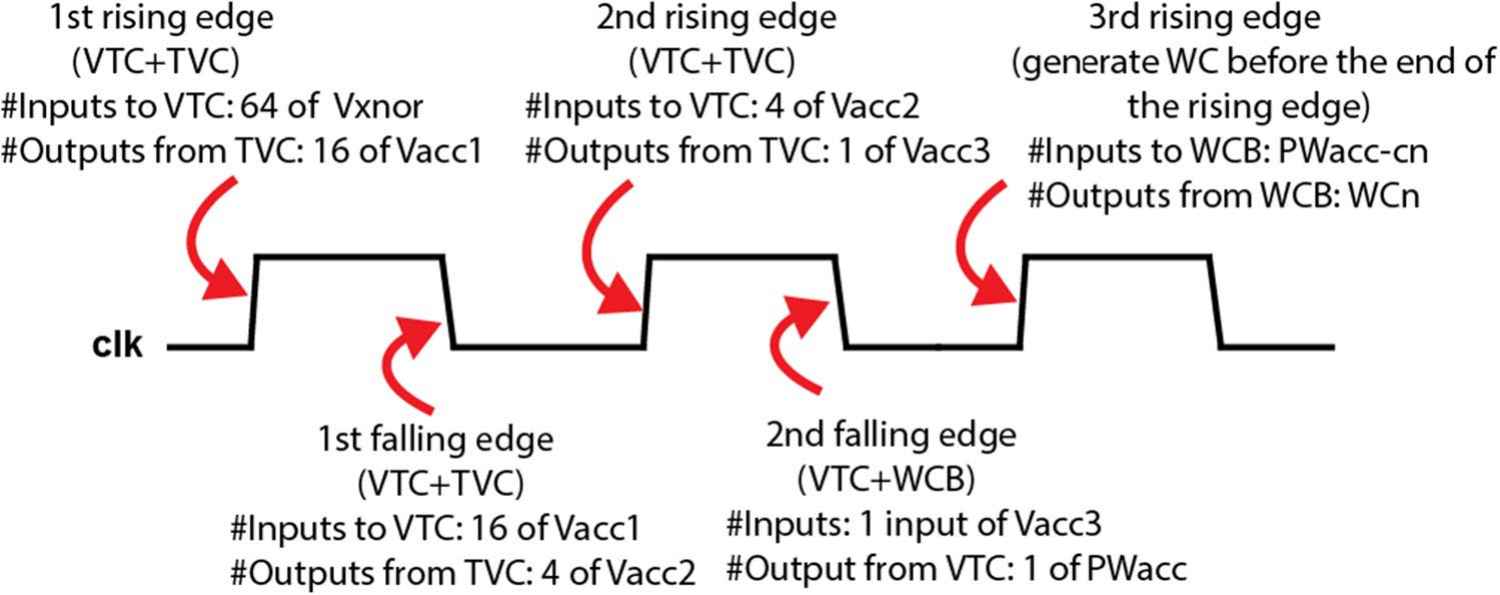
Timing diagram showing the input/output from each module of the full hierarchy of the mixed-signal XNOR-based RRAM-CAM at each rising edge.

The work proposed in this paper focuses on the physical implementation of XNOR-based RRAM-CAM for HDC classification. Nonetheless, in our paper^23^, simulations for both encoding/training and testing/inference phases for MNIST data-set were carried out. The effect of training data-set size, partial training, and chosen dimension *d* on the classification accuracy was studied. In the following section, a detailed step-by-step from applying the $1\times 1$ k query vector at the terminals of the XNOR-RRAM CAM until obtaining the winning class is shown in Fig. 7. And the subsequent logic used with the Hamming distance computations to obtain the winning class.

## Experimental section

### Proposed mixed-signal XNOR-based RRAM-CAM for HDC classification

In our proposed work, XNOR-RRAM CAM was used to perform the similarity check required for the Hamming distance computations. To be able to accommodate the hypervectors of $d = 1$ k for MNIST classification on the HDC paradigm, the RRAM-CAM is divided into 64 blocks each of $10\times 16$ as demonstrated in Fig. 7a. The sub-array has ten rows for the 10 MNIST classes and can tolerate 16 pairs of data with a noise margin of 30 mV. Resistance values of 1 M${\Omega }$ and 50 k${\Omega }$ for ‘R$_{OFF}$’ and ‘R$_{ON}$’, respectively, are adopted in the design and taken from real electric characterizations. A VTC follows each $10\times 16$ array to produce an output pulse representing the similarity between each of the 16 query pairs across the ten classes. All circuits were designed in 65 nm CMOS foundry and simulated using Cadence. After that, output pulse widths from 4 blocks are combined through the time-to-voltage interface shown in Fig. 7b. This is followed by another two combining stages in order to produce a single pulse for each class. Then, the ten pulse widths are passed to a winning class logic module, where each pulse corresponds to a single class set that consists of 4 OR gates and ten flip-flops to generate the winning class by determining the class with the longest pulse width as illustrated in Fig. 7c. Details of the individual design components are thoroughly discussed in Section Results.

The total time it takes to fully complete a single search task is two clock cycles which is 20 ns for a hypervector d = 1 kbit as shown in Fig. 8. At the 1st rising edge, every four cells of 16-bit XNOR-based RRAM are followed by VTCs. The four pulse widths are accumulated using TVC in parallel. The number of outputs from the accumulator is 16 voltage levels of Vacc1. At the 1st falling edge, each accumulated voltage level is converted again into pulses using VTC blocks. Then, every four pulses are accumulated using TVC. The output will be four accumulated voltage levels of Vacc2. At the 2nd rising edge, every voltage is converted into pulse width signal using VTC and then combined using TVC resulting in a single output voltage of Vacc3. At the 2nd falling edge, the single output voltage is followed by the VTC to generate the final pulse width signal pwacc. After that, once the WCB receives the signal from the 10 classes, it will generate the winning class before the 3rd rising edge. Thus, 2 clock cycles are needed. Monte Carlo simulations have been carried out for the end-to-end architecture starting from the RRAM-CAM till the winning class block for two classes and the results are illustrated in Table S4.

In order to evaluate the advantages of the proposed implementation of the proposed design, we compare it with other works in terms of area and energy. The estimated area calculation for the RRAM-based CAM is based on a fabricated full-pitch width of 400-nm from^24^. The full CAM is divided into 64 blocks, each with a dimension of $10\times 16$. Remember that 16 pairs of memristors mean 32 devices. This occupies an area of [64 $\times$ (400 nm $\times$ 16 $\times$ 2) $\times$ (400 nm $\times$ 10)] = 0.0032768 mm${^2}$ that will accommodate 1024 bits. The measured area for the VTC, TVC, MUXES, and winning class logic through cadence was 0.0047 mm${^2}$ in 65 nm CMOS technology. As a result, the proposed design’s total area is 0.0077 mm${^2}$. Table 1 demonstrates the comparison between the main designs in the literature and the work presented in this paper. Area scaling was obtained through^25^.

**Table 1:** Energy and area metrics of our proposed time-domain RRAM-CAM HAM design and D-HAM compared to references normalized to: 65 nm, $d=1$ k, and 10 Classes.

Metrics	Ref^13^	Ref^19^			This work	
Total area (mm${^2}$)	0.0343	D-HAM 0.1723	R-HAM 0.1230	A-HAM 0.0574	Proposed 0.0077	D-HAM 0.237
Energy per query (1 k) (pJ)	579.1	D-HAM 61.546	R-HAM 12.589	A-HAM *	Proposed 13.6	D-HAM 42.9

Energy for the proposed design is estimated from Cadence SPICE simulation for all components from Fig. 2c = 13.6 pJ. The activity factor for the RRAM crossbar is 0.5 since the distances from any arbitrary chosen HD-vector to another one is around 0.5 normalized Hamming distance^26^. While the activity factor for the other circuits of VTC, TVC, and WCL is 1 since they are dynamic, charge and discharge in very cycle. It is worth mentioning that the write time and energy were not included in the reported values as they occur only once and the values are then fixed throughout the lifetime of the device. Also, the compute voltage for the RRAM-CAM crossbar is below the write voltage of the devices to eliminate any state disturb.

Results show a remarkable reduction in area and energy compared to the state-of-the-art RRAM designs. *Energy for the Analog-HAM design reported in^19^ was not included in the Table as no reliable data can be extracted on energy. For example, compared to the PCM-based AM in^13^, our design exhibits a $\sim$ 4.5 $\times$ reduction in area and $\sim$ 42.6 $\times$ lower energy consumption.

### ASIC hyperdimensional associative memory

**Figure 9:**
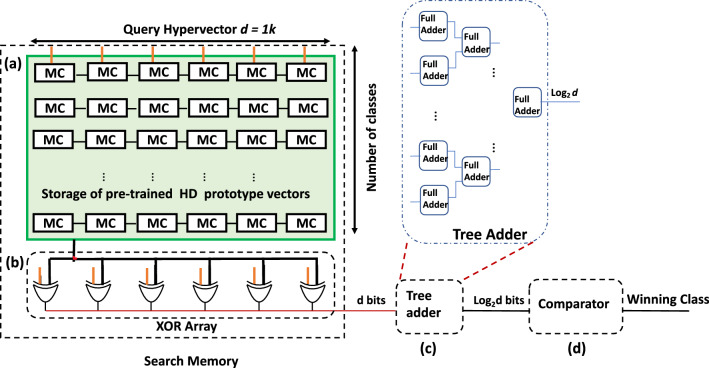
Sequential implementation for the digital hyperdimensional associative memory. The design includes the following modules: (**a**) array of memory cells (MC) of size 1 k $\times$ 10, where 1 k bits is the assumed vector dimension, and 10 is the number of stored classes. (**b**) An array of 1024 XOR gates, (**c**) a tree adder, and (**d**) a comparator.

Digital ASIC hyperdimensional AM is implemented using 65 nm CMOS foundry technology and a proven tape-out design flow based on Synopsys tool suites, including ICC2. Figure 9 illustrates the block diagram of the digital ASIC hyperdimensional AM. It consists of an array of ($P \times d$) latches or storage elements, where *P* refers to the number of prototype classes and *d* to the HD vector dimension. A vector of 1024 XOR gates to perform the comparison between the encoded input and pre-stored encoded data. The tree of binary adders consists of adders’ stages and has a depth of $\log _{2}$ d. In each stage j, where j ranges from [1, $\log$ d], the adder’s width is j bits, and the number of adders involved in the addition operation is $d^j/2$. For example, to sum the number of ones in a 1k bits vector, the first stage deploys 512 bit-wise adders, while the second stage has 256 2-bits adders. The tree adders eventually result in a 10-bit output that depicts the number of ones in the vector. The adders’ output contains the value of Hamming distance between the query HD vector and the corresponding stored HD class in that row. A digital comparator is used to find the minimum Hamming distance value received from the tree adders. Detailed place and route design for the 1 k vector dimension is implemented. The design structure is scalable and can be extended to higher dimensions. The area for digital hyperdimensional associative memory configured for *P* =10 and *d* = 1024. That requires the array of storage elements to be of dimension $10\times 1024$ along with XOR row of 1024 gates, 1022 number of adders, and a comparator of 10 bits. Using a sequential design to compute the Hamming distance that shares the same resources of XOR gates and comparators results in $O(n_{classes})$ of time complexity that depends on the number of available classes. So for the case of MNIST classification with 10 output classes; $O(n_{classes})$ = 10 cycles. Sharing resources take advantage of reducing the area at the cost of a long clock cycle.

**Figure 10:**
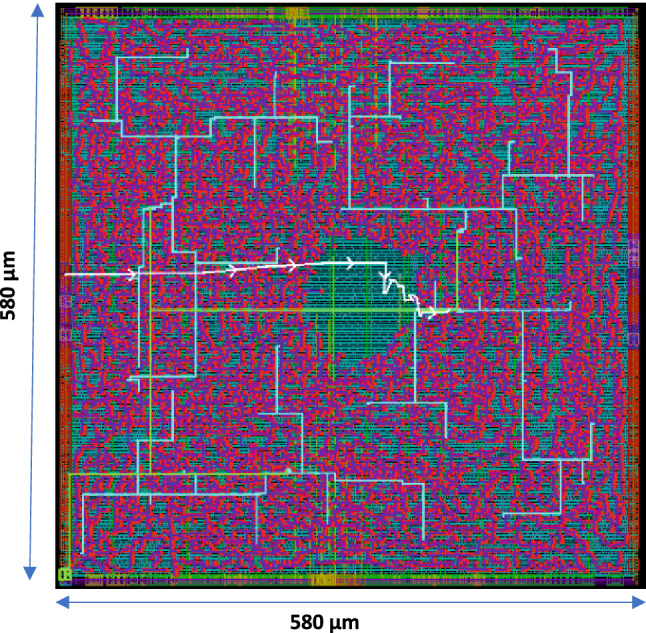
The layout of the ASIC-flow design for the digital AM. It includes the chip area utilization and the critical path.

The ASIC design for 65% area utilization results in a total area of 0.237 mm$^2$, while the energy reported for the 1 k query search and $V_{dd}$ = 1.08 V is 42.9 pJ with a cycle time of 10 ns (100 Mhz). The data is reported using a regular threshold voltage CMOS transistor. The chip layout and the critical path are depicted in Fig. 10. So far, the state-of-the-art ASIC implementation for Hamming distance in HDC^27–29^ counts the number of match/mismatch using a binary counter that passes through all vector elements. Though this implementation seems hardware friendly, the latency overhead would reach a time complexity of *O*(*d*) cycles. Referring to Table 1, a reduction of $\sim$ 31$\times$, $\sim$ 3$\times$ in area and energy is obtained when utilizing the proposed XNOR-based RRAM-CAM with time-domain analog adder instead of the digital ASIC counterpart.

## Conclusion

In this paper, an XNOR-based RRAM-CAM with a time-domain analog adder for efficient winning class computation is proposed. The design consists of three main blocks: XNOR-based RRAM-CAM, time-domain adder, and a winning class logic. The CAM takes one operand in voltage and the second in resistance and outputs a voltage proportional to the similarity between the input query and pre-stored patterns. The output voltage of XNOR is translated into pulse width via VTCs and TVCs. Eventually, to determine the winning class among the multiple classes, the digital block is utilized to consider the class with the longest pulse width as the winner. Many critical domain applications require fast search engines with high performance to processes large amounts of input queries. Hence, HDC for efficient MNIST classification is considered as it requires performing a search in thousands of bits query length.

The proposed mixed-signal XNOR-based RRAM-CAM approach for HDC classification provides a significant savings of $\sim$ 31 $\times$, $\sim$ 3 $\times$ in area and energy respectively compared to the digital ASIC approach. Also, the proposed design exhibits a remarkable reduction in area and energy compared to the state-of-the-art RRAM designs.

In the future, efforts will focus on implementing the encoding process consisting of addition, multiplication, and cyclic shifting operations using hardware-based IMC designs. This will pave the way to implementing efficient solutions compared to current approaches.

## Supplementary Information

Supplementary Information.

## References

[1] de Lima JPC, de Moura RF, Carro L (2020). Analog memristive CAMs for area-and energy-efficient reconfigurable computing. IEEE Trans. Circuits Syst. II Exp. Briefs.

[2] Halawani Y, Mohammad B, Lebdeh MA, Al-Qutayri M, Al-Sarawi SF (2019). ReRAM-based in-memory computing for search engine and neural network applications. IEEE J. Emerg. Select. Topics Circuits Syst..

[3] Halawani Y, Lebdeh MA, Mohammad B, Al-Qutayri M, Al-Sarawi SF (2018). Stateful memristor-based search architecture. IEEE Trans. Very Large Scale Integr. (VLSI) Syst..

[4] Kaplan R, Yavits L, Ginosar R (2018). RASSA: Resistive prealignment accelerator for approximate DNA long read mapping. IEEE Micro.

[5] Mohammad K, Qaroush A, Washha M, Mohammad B (2017). Low-power content addressable memory (cam) array for mobile devices. Microelectron. J..

[6] Mohammad, B., Bassett, P., Abraham, J. & Aziz, A. Cache organization for embeded processors: cam-vs-sram. *In IEEE International SOC Conference* **299–302**, (2006).

[7] Mahendra TV, Mishra S, Dandapat A (2017). Self-controlled high-performance precharge-free content-addressable memory. IEEE Trans. Very Large Scale Integr. (VLSI) Syst..

[8] Xie, L. *et al.* Scouting logic: A novel memristor-based logic design for resistive computing. *IEEE Computer Society Annual Symposium on VLSI (ISVLSI)* **176–181**, (2017).

[9] TaheriNejad N (2021). Sixor: Single-cycle in-memristor xor. IEEE Trans. Very Large Scale Integr. (VLSI) Syst..

[10] Rajaei R, Sharifi MM, Kazemi A, Niemier M, Hu XS (2020). Compact single-phase-search multistate content-addressable memory design using one FeFET/cell. IEEE Trans. Electron Dev..

[11] Li C (2020). Analog content-addressable memories with memristors. Nat. Commun..

[12] Park HK, Ahn HK, Jung S-O (2020). A novel matchline scheduling method for low-power and reliable search operation in cross-point-array nonvolatile ternary CAM. IEEE Trans. Very Large Scale Integr. Syst..

[13] Karunaratne, G. *et al.* In-memory hyperdimensional computing. *Nat. Electron.* **1–11**, (2020).

[14] Taha MM, Teuscher C (2020). Approximate memristive in-memory Hamming distance circuit. ACM J. Emerg. Technol. Comput. Syst..

[15] Vranesic ZG, Brown S (2000). Fundamentals of Digital Logic with VHDL Design.

[16] Murshed, M. *et al.* Machine learning at the network edge: A survey. arXiv:1908.00080 (2019).

[17] Humood, K. *et al.* High-density reram crossbar with selector device for sneak path reduction. In *2019 31st International Conference on Microelectronics (ICM)*, 244–248 (IEEE, 2019).

[18] Srivastava S, Dey P, Asapu S, Maiti T (2018). Role of GO and r-GO in resistance switching behavior of bilayer TiO$$_{2}$$ based RRAM. Nanotechnology.

[19] Imani, M., Rahimi, A., Kong, D., Rosing, T. & Rabaey, J. M. Exploring hyperdimensional associative memory. *In IEEE International Symposium on High Performance Computer Architecture (HPCA)* **445–456**, (2017).

[20] Naraghi, S. *Time-Based Analog to Digital Converters.* Ph.D. thesis (2009).

[21] Räsänen O, Kakouros S (2014). Modeling dependencies in multiple parallel data streams with hyperdimensional computing. IEEE Signal Process. Lett..

[22] Ge L, Parhi KK (2020). Classification using hyperdimensional computing: A review. IEEE Circuits Syst. Magazine.

[23] Hassan, E., Halawani, Y., Mohammad, B. & Saleh, H. Hyper-dimensional computing challenges and opportunities for ai applications. *IEEE Access* (2021).

[24] Sheridan PM, Du C, Lu WD (2016). Feature extraction using memristor networks. IEEE Trans. Neural Netw. Learn. Syst..

[25] Stillmaker A, Baas B (2017). Scaling equations for the accurate prediction of CMOS device performance from 180 nm to 7 nm. Integration.

[26] Kanerva P (2009). Hyperdimensional computing: An introduction to computing in distributed representation with high-dimensional random vectors. Cognit. Comput..

[27] Imani, M., Kong, D., Rahimi, A. & Rosing, T. Voicehd: Hyperdimensional computing for efficient speech recognition. *IEEE International Conference on Rebooting Computing (ICRC)* **1–8**, (2017).

[28] Imani, M., Rahimi, A., Kong, D., Rosing, T. & Rabaey, J. M. Exploring Hyperdimensional Associative Memory. *In Proceedings - International Symposium on High-Performance Computer Architecture* **445–456**, (2017).

[29] Rahimi A, Kanerva P, Benini L, Rabaey JM (2018). Efficient biosignal processing using hyperdimensional computing: Network templates for combined learning and classification of ExG signals. Proc. IEEE.

