# Supplementary material for: RRAM-based CAM combined with time-domain circuits for hyperdimensional computing
Source: Sci Rep. 2021 Oct 6;11:19848. doi: 10.1038/s41598-021-99000-w (PMC8494892; doi:10.1038/s41598-021-99000-w)
Supplement: Supplementary file 1 — Supplementary Information. [file 41598_2021_99000_MOESM1_ESM.docx]

**Supplementary Information**

**RRAM-Based CAM Combined with Time-Domain**

**Circuits for Hyperdimensional Computing**

Yasmin Halawani^1^, Dima Kilani^1^, Eman Hassan^1^, Huruy Tesfai^1^, Hani Saleh^1^, and Baker

Mohammad^1, *^

^1^System-on-Chip Center (SoCC), Department of Electrical and Computer Engineering, Khalifa University, Abu Dhabi, UAE

[^*^baker.mohammad@ku.ac.ae](mailto:*baker.mohammad@ku.ac.ae)

**Supplementary Figures**


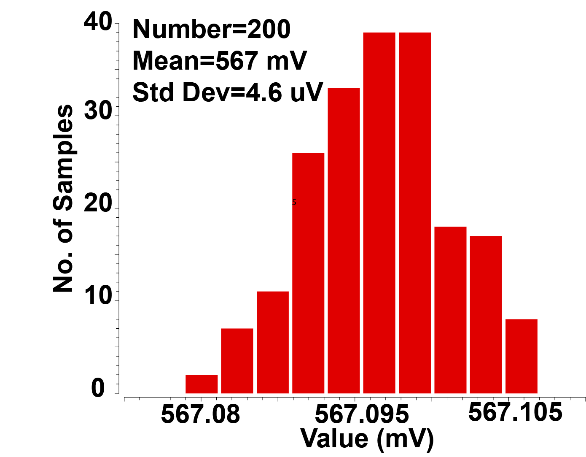


**Figure S1.** 16-bit XNOR-based RRAM histogram mismatch variations for 16 matching-input cells. The x-axis represents the V_XNOR_ value and y-axis represents the number of samples.


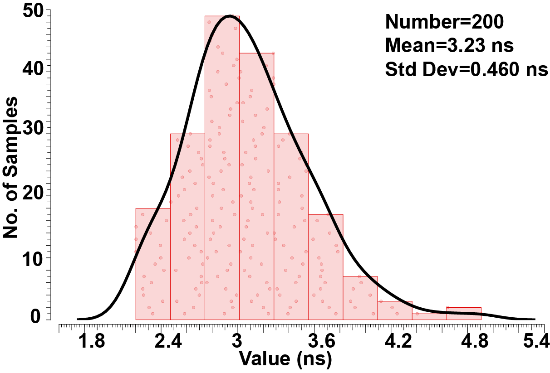


**Figure S2.** Histogram mismatch variations on pw from VTC block.


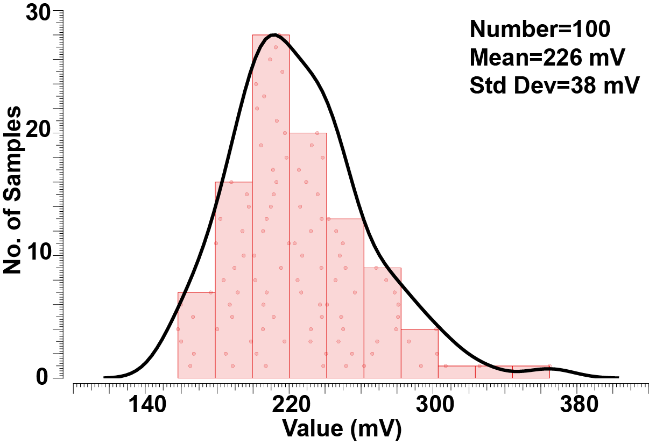


**Figure S3.** Histogram mismatch variations on accumulated voltage level Vacc from 32-bit XNOR-based RRAM cells.


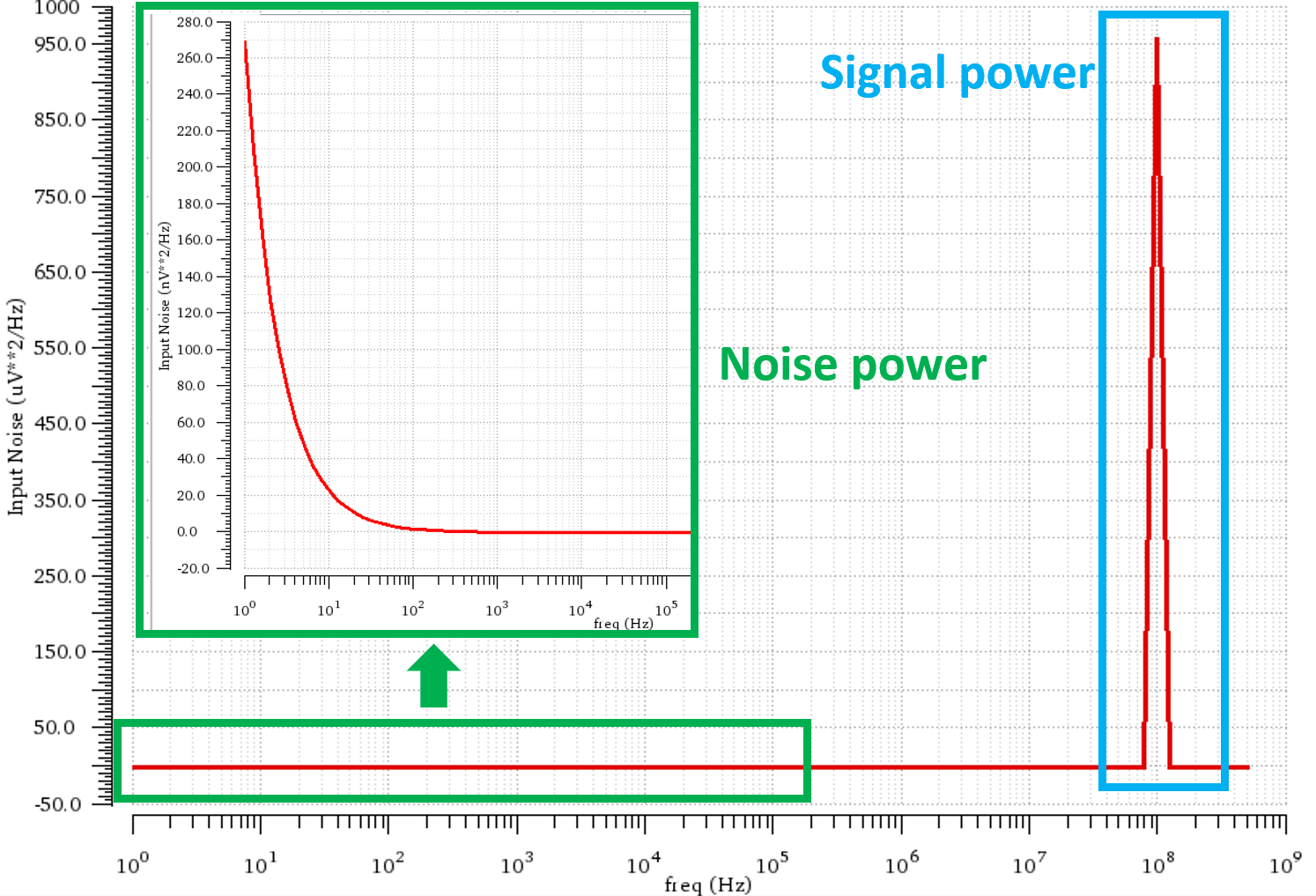


**Figure S4.** Power spectrum of the input-referred noise in the VTC at V_XNOR_=0.567V and clock frequency=100MHz.

**Supplementary Tables**

**Table S1.** Memristor VTEAM Model specifications

| **Parameter** | **Value** |
| --- | --- |
| **Model** | 4 |
| **Window_type** | 0 |
| **dt** | 1e-3 |
| **Init_state** | 0 for low (50k) and 1 for high (1M) |
| **Roff** | 50k |
| **Ron** | 1M |
| **D** | 125e-9 |
| **K_on** | -30 |
| **K_off** | 7.67e-2 |
| **Alpha_on** | 2.887 |
| **Alpha_off** | 3 |
| **V_on** | -1.2 |
| **V_off** | 0.6 |
| **IV_relation** | 0 |
| **X_on** | 0 |

**Table S2.** Trade-off between the number of pairs in a XNOR-Based RRAM and the corresponding noise margin between successive matching and mismatching cells, in addition to the current consumption

|  | **8 pairs** | **16 pairs** | **32 pairs** |
| --- | --- | --- | --- |
| **Delta (mV)** | 80.58 | 39.89 | 15.68 |
| **Current (max) (uA)** | 19.16 | 39.17 | 76.85 |

**Table S3.** Variations for different number of VTC stages

| **Number of VTC stages** | **Mean (ns)** | **Standard deviation (ns)** | **Variation (%)** |
| --- | --- | --- | --- |
| 1 | 3.23 | 0.460 | 14.2 |
| 2 | 6.44 | 0.656 | 10.2 |
| 3 | 12.29 | 0.817 | 6.6 |
| 4 | 15.04 | 0.905 | 6 |

**Table S4.** Impact of mismatch variations on the error percentage for class 1 and class 2 over 100 samples when the number of bit difference between the two classes varies

| #**bit difference between class 1 and class 2** | **Error percentage of WC1** | **WC2 percentage of WC2** |
| --- | --- | --- |
| 2 | 5% | 28.9% |
| 3 | 2% | 15.8% |
| 4 | 0% | 8.4% |

**Supplementary Notes**

Figure S1. shows the XNOR-based RRAM histogram mismatch variations when all 16 XNOR cells are input-matched and RON and ROFF values are varied by +10%. The standard deviation of 4.6 mV at mean value of 0.567 V.

Figures S2 and S3. show the effect of mismatch variations on the pw of VTC obtained from Monte Carlo simulation on the time delay for 200 samples. As depicted from the figure S2., the standard deviation has a low value of 0.481 ns from the mean of 3.01 ns. Hence, the ratio of the standard deviation to the mean is approximately 14.2%. This variation can be reduced by cascading multiple stages of the VTC circuit. Simulation results shows that for 4-stages VTC, the variation decreases down to 6%. This is demonstrated in Table S3.

Figure S3 depicts the effect of mismatch variations on the accumulated voltage Vacc from the time-based adder (both VTC and TVC blocks). As depicted from the figure, the standard deviation is 38 mV from the mean of 226 mV. Hence, the ratio of the standard deviation to the mean is approximately 16.8%.

Noise simulation, in Figure S4, has been carried out to analyze the input-referred noise and the SNR of the time-based analog adder whose input is Vxnor and output is pwacc. The Vxnor value is set at 0.567 V when the 16 XNOR cells are input-matched and the clock frequency= 100 MHz. The input noise and signal power averages are obtained by integrating the noise and signal power spectrums over their frequency range. Spice simulation shows that the averaged input referred-noise and signal are 30 uV^2^ and 22.2 mV^2^ resulting in an SNR value of 28.7 dB.

Tables S1 provides the VTEAM model specifications used in our crossbar simulations. And Table S2 demonstrates the trade-offs between the number of memristor devices, noise margin and current consumption.

In order to study the impact of the mismatch variation on the accuracy of the proposed time-domain RRAM-CAM Hamming distance and winning class, Monte Carlo simulations have been carried out for the end-to-end architecture starting from the RRAM model till the winning class block for two classes. Table S4 shows the difference error percentage over 100 samples for class 1 WC1 and class 2 WC2 when the bit difference is 2, 3 and 4. As shown from the table, the 2-bit difference is the worst-case scenario with an error of 31% where the hamming distance for the two classes are quite close. As the number of bit difference between the two classes increases, the error of the winning class logic signal decreases. Note that in our simulation, the number of bit is kept fixed for class 2 and increased by class 1. Although the error percentage is high for the worst-case scenario, HDC is tolerable to errors due to its holographic representation where bits are independent of their positions.
